# Relationship Between Thyroid Hormonal Status in Patients with a Hypothyroid Form of Hashimoto’s Thyroiditis and Iodine Concentrations in Drinking Water

**DOI:** 10.1007/s12011-021-02640-2

**Published:** 2021-03-02

**Authors:** Olha Kasiyan, Halyna Tkachenko, Natalia Kurhaluk, Svitlana Yurchenko, Alek Manenko

**Affiliations:** 1 Danylo Halytsky Lviv National Medical University, Lviv, Ukraine; 2 Institute of Biology and Earth Sciences, Pomeranian University in Słupsk, Arciszewski Str. 22b, 76-200 Słupsk, Poland; 3 Lviv Medical Institute, Lviv, Ukraine

**Keywords:** Hashimoto’s thyroiditis, Endemic region, Water iodine concentration, Thyroid-stimulating hormone, Thyroglobulin antibodies, Thyroxine peroxidase

## Abstract

The current study aimed to identify correlative and regressive dependencies between the water iodine concentration and the levels of TSH (thyroid-stimulating hormone), thyroglobulin antibodies (TgAbs), and thyroid peroxidase (TPOAb) in the serum of 168 in patients (34 men and 134 women) with a hypothyroid form of Hashimoto’s thyroiditis who use water from the supply network and individual wells. Based on the water iodine concentration, low and moderate degrees of iodine endemia in the location of the patients were determined. In the groups of men and women using water from different water supply sources, there were direct correlations between the water iodine concentrations and the TgAbs and TPOAb titers as well as an inverse dependence between iodine and TSH levels. Multivariate regressive analysis indicated that TgAb and TSH in the group of women using water from a supply network and TPOAb titers in the group of women using well water were independent factors associated with water iodine concentrations. Statistically significant correlations and regressive dependencies between the water iodine concentrations and the biomarkers of the thyroid status of the patients indicate the risk of Hashimoto’s thyroiditis progression, especially among women with additional iodine intake.

## Introduction

Hashimoto’s thyroiditis (HT) is one of the most prevalent autoimmune diseases leading to the formation of anti-thyroid antibodies that attack the thyroid tissue, causing lymphocyte-mediated cell-damaging process and leading to the destruction of follicular cells and progressive fibrosis [11, 30]. The current diagnosis of Hashimoto’s disease is based on clinical symptoms correlated with laboratory results showing elevated thyroid-stimulating hormone (TSH) with normal to low thyroxin levels [29]. The role of anti-thyroid peroxidase (anti-TPO) antibodies in the pathogenesis of autoimmune thyroid disease (AITD) is negligible. There has been no correlation noted in human studies between the severity of the disease and the level of anti-TPO antibody concentration in serum [45]. The pathogenetic mechanisms leading to the development of autoimmune thyroid disease are based on several different factors [36]. It is well established that 20% of etiology is attributed to environmental factors (smoking, iodine intake, selenium deficiency, pollution, infectious conditions, physical and emotional stress) and physiological states (puberty, rapid growth, pregnancy, menopause, aging, female sex) [2, 41]. There is also increasing evidence that mainly nutritive factors and environmental pollution by metals and chemicals are the main factors in the present-day spread of AITD [5]. Moreover, the contribution of each factor varies from patient to patient, and there are no clear genotype-phenotype correlations [21].

Iodine is an essential trace mineral required for the production of the thyroid hormone [33]. The thyroid gland is an effective collector of iodine and has several protective mechanisms resulting in the maintenance of normal thyroid function despite wide fluctuations of the daily iodine intake. In the presence of defective auto-protective mechanisms, excessive iodine ingestion can divert the normal thyroid function [19]. Hypothyroidism or hyperthyroidism as a result of supraphysiologic iodine exposure may be either subclinical or overt, and the source of the excess iodine may not be readily apparent [25]. Iodine is often considered to be one of the trigger factors in the development of autoimmune thyroiditis. An excess of iodine can cause or contribute to the progression of the autoimmune process in the thyroid tissue increasing the level of intra-thyroid infiltrating Th17 cells and inhibiting T regulatory (TREG) cell development. It also triggers abnormal expression of tumor necrosis factor-related apoptosis-inducing ligand (TRAIL) in thyrocytes, thus inducing apoptosis and destruction of the thyroid gland parenchyma [6].

Iodine in drinking water has been used as an indicator of the “iodine state” of an area; thus, iodine in drinking water may also be a major direct source of iodine intake, which may determine regional variations in iodine intake levels [34]. People living permanently in an area where the soil is iodine deficient and where all food originates from such soil are at risk of developing iodine deficiency diseases [44]. Even though iodine is present in all components of the environment, water has been considered an indicator of the iodine supply in a certain area. Water, passing its cycle, dissolves chemicals in the soil and takes the so-called average sample from everything in contact. The iodine content in the soil depends on its type. From the soil, iodine enters groundwater and then the human body with the drinking water supply. Levels of iodine in drinking water supplies are reflective of such factors as iodine in the soil and water table, proximity to seawater, and agricultural runoff [7, 9]. As a result, the iodine content in water can indicate the possibility of IDD in a particular area. The iodine-induced endemia is determined with the use of the water iodine concentration, i.e., an iodine concentration up to 2 μg/dm^3^ confirms its high degree, 2–3 μg/dm^3^ (moderate level) and 3–4 μg/dm^3^ (weak iodine-induced endemia) [31].

Ukraine is one of the few countries in Europe where the problem of prevention of iodine deficiency diseases has not been solved even though it has been included in the programs of UNICEF and the International Committee for the Control of Iodine Deficiency Diseases (ICCIDD). The geological features of the territory of Ukraine determine the presence of iodine deficiency in the western and northern regions of the country. The presence of iodine deficiency of varying degrees has been demonstrated in almost all of Ukraine [20, 42]. In Ukraine, areas with partial iodine deficiency, with moderate and mild iodine deficiency, and regions with sufficient iodine availability have been identified [20]. The Lviv region in Ukraine, along with Volyn, Rivne, Ternopil, Ivano-Frankivsk, Zakarpattia, and Chernivtsi regions, is one of the areas with a high degree of endemic iodine deficiency. An unequal degree of iodine deficiency within the Lviv endemic region based on the iodine content in the drinking water of settlements has also been revealed [14, 16]. Currently, iodine deficiency is observed in many other regions as well (Donetsk, Sumy, Zhytomyr, Kyiv, Chernihiv, Luhansk, Republic of Crimea) [20]. Since not the whole territory of Ukraine is iodine deficient and an unequal degree of iodine deficiency is recorded even in the same region, the approach to the prevention of iodine-dependent diseases in different regions should be differentiated. The determination of correlations between the water iodine concentration and cases of HT will provide an opportunity for more differentiated iodine prophylaxis including iodine intake by inhabitants in each region.

Therefore, the aim of the current study was (i) to assess the levels of the thyroid-stimulating hormone and antibodies to thyroglobulin and thyroid peroxidase in patients with a hypothyroid form of Hashimoto’s thyroiditis of both sexes, (ii) to collect drinking water from the water supply network and individual wells of these patients, and (iii) to identify correlations between the water iodine concentration in water and these indicators in patients using drinking water from the water supply network and individual wells.

## Materials and Methods

### Subjects

An analysis of case histories from 2182 patients from the Lviv region (western region of Ukraine) who underwent inpatient treatment at the Lviv Regional Endocrinology Dispensary was carried out in this study. Case histories of 168 patients with diagnosed Hashimoto’s thyroiditis (hypothyroid form) were selected for the current study. The registration address of these patients in the case histories did not differ from their current place of residence. In total, 168 individuals, i.e., 34 males and 134 females with a mean age of 33.98 ± 3.51 and 41.55 ± 1.43 years, respectively, participated in this study. In this group of patients, 53 females (31.6%) and 15 males (8.9%) consumed water from the water supply network, while 81 females (48.2%) and 19 males (11.3%) consumed water from individual wells. The females and males who consumed water from the water supply network and the individual wells did not differ in age, i.e., females: 41.70 ± 1.46 vs. 41.40 ± 1.40 years (*p* = 0.880) and males: 35.67 ± 3.64 *vs.* 31.58 ± 3.40 years (*p* = 0.421), respectively. Individuals with malignancies and thyroid surgery were excluded from the study.

### Analysis

Thyroid hormone status was assessed by determination of the concentrations of TSH (thyroid-stimulating hormone), thyroglobulin antibodies (TgAbs), and thyroid peroxidase (TPOAb) in serum. The concentration of TSH was measured by radioimmunoassay analysis, while immunochemiluminescence assays were used for TgAbs and TPOAb concentrations. All analytical procedures were carried out at the Clinical Laboratory of Regional Clinical Hospital in Lviv. The reference ranges were as follows: 0.4–4 mIU/L for TSH, < 115 IU/L for TgAb, and < 34 IU/L for TPOAb [38].

Determination of the iodine concentration in water was carried out according to the “Method for measurements of the iodine concentration in drinking and mineral water, salt, bakery products, dairy products by the method of inversion voltammetry on a solid rotating electrode No. 081/12-0092-03” at the Lviv regional sanitary-hygienic laboratory using a mercury-film electrode and an AVA-2 device [15, 28]. Samples of drinking water were collected from patients’ home addresses: 64 samples from the centralized water supply systems and 104 samples from the individual wells. All sample measurements were made in duplicates and re-analyzed if the duplicates differed by more than 2% in absorbance.

The present study is a fragment of the research project “Hygienic evaluation of the joint influence of iodine insufficiency and other factors on the health welfare of children’s population” (state registration No 0108 U001136). The Ethics Committee of Danylo Halytsky Lviv National Medical University (Lviv, Ukraine) approved the study protocol (No. 13 of October 20, 2005). All participants gave informed consent before blood and water sampling.

### Statistical Analysis

The procedure for the analysis of the normality of all samples using the Shapiro-Wilk criteria was performed for statistical data analysis. The mean (M) and the standard error of the mean (m) value were calculated. Since the data were not normally distributed, the differences between the groups were tested using the Mann-Whitney test. The correlations between the studied parameters in the groups were evaluated using Spearman’s rank correlations, while multivariate stepwise regression analysis was used for regression analysis. The critical level of statistical significance (p) was assumed to be 0.05 [46]. The initial preparation of intermediate calculations was performed using Microsoft Excel 13.0. The obtained results were statistically analyzed using the STATISTICA 8.0 software package (StatSoft, Krakow, Poland).

## Results

The water iodine concentration in the water supply network and individual wells indicates a weak and moderate degree of iodine endemia in the studied areas (Table 1). The iodine concentrations differed only in the water consumed by the men: the iodine concentration in the water supply network was 1.53-fold lower (*p* = 0.024). Statistical differences were also observed between the values of the iodine concentration in the network water, which was consumed by both males and females (*p* = 0.017).

**Table 1:** The mean water iodine concentration in drinking water used by male and female patients with a hypothyroid form of Hashimoto’s thyroiditis

Groups of patients	Water iodine concentration (M ± m), μg/dm^3^				p
	*n*	Water supply network	*n*	Individual wells	
Females	53	3.02 ± 0.19	81	3.20 ± 0.15	0.472
Males	15	2.13 ± 0.31	19	3.25 ± 0.35	0.024
p	0.017	0.892			

No significant differences were found between the mean TgAb and TPOAb titers and the TSH levels in females who consumed drinking water from the water supply network and individual wells (Table 2). In males drinking water from different water supply sources, only TgAbs titers differed significantly (45.60 ± 3.69 *vs*. 61.26 ± 3.87 IU/L, *p* = 0.006).

**Table 2:** Biomarkers of hormonal thyroid status of male and female patients with a hypothyroid form of Hashimoto’s thyroiditis

Groups of patients	TgAb, IU/L	TPOAb, IU/L	TSH, mU/L
Females			
Water supply network	60.85 ± 3.90	51.26 ± 2.68	12.72 ± 2.15
Individual wells	57.96 ± 2.93	52.80 ± 2.21	15.02 ± 1.73
*p*	0.485	0.769	0.153
Males			
Water supply network	45.60 ± 3.69	50.13 ± 3.11	9.62 ± 3.14
Individual wells	61.26 ± 3.87	54.68 ± 4.21	10.31 ± 3.0
*p*	0.006	0.391	0.868
All patients	58.14 ± 1.97	52.29 ± 1.46	13.38 ± 1.17

Also, an increase in the water iodine concentration resulted in an increment in the TgAb titers in both groups of females and males consuming water from the water supply network and in the TPOAb levels in the female group drinking water from the individual wells (Table 3). An inverse correlation between the water iodine concentrations and TSH levels in female patients consuming water from the water supply network was recorded. These dependencies were moderate.

**Table 3:** Correlations between the thyroid hormone status in patients with a hypothyroid form of Hashimoto’s thyroiditis and water iodine concentrations

Groups of patients	Correlation coefficients		
	TgAb	TPOAb	TSH
Females			
Water supply network	0.348, *р* = 0.014	0.220, *p* = 0.129	−0.584, *p* < 0.001
Individual wells	0.109, *p* = 0.321	0.343, *p* < 0.001	0.057, *p* = 0.610
Males			
Water supply network	0.564, *p* = 0.028	−0.035, *p* = 0.901	−0.053, *p* = 0.871
Individual wells	0.296, *p* = 0.218	0.426, *p* = 0.068	0.397, *p* = 0.102
All patients	0.237, *p* = 0.002	0.305, *p* < 0.001	−0.028, *p* = 0.733

The results of the multivariate regression analysis suggested that the TgAb and TSH levels in the group of females consuming water from the water supply network were independent of the water iodine concentrations (Table 4). In the group of females who consumed water from the individual wells, the TPOAb level was this factor. No such independent factors were identified in the group of males consuming water from the water networks and wells. Among both female and male patients, TgAb and TPOAb were independent factors associated with the iodine concentration in water (Table 4).

**Table 4:** Regressive analysis of relationships between thyroid hormone status in patients with a hypothyroid form of Hashimoto’s thyroiditis and water iodine concentrations

Groups of patients	Regression coefficients		
	TgAb	TPOAb	TSH
Females			
Water supply network	0.017, *p* = 0.005	0.011, *р* = 0.164	−0.046, *р* < 0.001
Individual wells	0.004, *р* = 0.497	0.017, *р* = 0.033	−0.002, *р* = 0.865
Males			
Water supply network	0.021, *р* = 0.172	0,029, *р* = 0,166	0.006, *р* = 0.762
Individual wells	0.037, *р* = 0.197	0.002, *р* = 0.953	0.037, *р* = 0.393
All patients	0.010, *р* = 0.02	0.016, *р* = 0.007	−0.008, *р* = 0.312

## Discussion

The results of this study indicate that, in endemic areas with mild and moderate iodine deficiency, there were correlations between the iodine concentrations in drinking water and the titers of antibodies to thyroglobulin and thyroid peroxidase (TgAbs and TPOAb) as well as the TSH levels in the serum of male and female patients with a hypothyroid form of Hashimoto’s thyroiditis, which indicated the risk of disease progression with additional iodine intake in the organism, especially among the women (Table 3). This assumption would be more justified by an analysis of the intake of iodine-containing foods and pharmaceuticals by the patients, which could be a direction for further research. It is hard to know to what extent iodine-containing foods originate from outside the study area. Since food consumption studies are also very time-consuming when carried out on an individual basis and provide only a “snapshot” impression of the actual intake, they are generally only carried out on a population basis, provided that dietary habits and the iodine content in consumed foods are reasonably well known. In that case, the average iodine intake of certain population groups may be estimated and these data together with clinical data may form the basis for decisions on policy measures in the field of salt iodization and the application of iodized salt in various processed foods [44].

Although iodine supplementation has decreased the number of individuals at risk of iodine deficiency and its associated sequelae, particularly in the past few decades, the use of iodine has also led to concerns of excessive iodine exposure in some individuals [25]. Partly reversible iodine-induced thyroid dysfunction and autoimmunity were observed among patients with endemic goiter [13]. For example, high iodine intake seems to increase the prevalence of autoimmune thyroiditis in the Bio-Breeding/Worcester rat model and humans. It has been suggested that the incidence of Hashimoto’s thyroiditis is increased in the presence of high iodine intake. The iodine intake significantly affects the incidence of spontaneous lymphocytic thyroiditis in genetically predisposed young rats [1].

Monitoring and adjustment of iodine intake in the population from biogeochemical provinces is an important part of preventive medicine. Several environmental factors influence the epidemiology of thyroid disorders, and even relatively small abnormalities and differences in the level of iodine intake in a population have profound effects on the occurrence of thyroid abnormalities [24]. Many data indicate that iodine supplementation in populations with low iodine intake can increase the incidence of autoimmune Hashimoto’s thyroiditis [24]. Thomson et al. [39] have investigated the effects of excess iodine intake as iodate on the thyroid and selenium status in New Zealand. Excess iodate induced hypothyroidism in some participants and hyperthyroidism in others [39].

Also, various correlative and regressive dependencies concerning TSH levels and water iodine concentrations in different groups of patients were observed (Tables 3 and 4). Probably, this was due to an increased iodine supply, which resulted in changes in the metabolism of thyroid hormones and, consequently, some decrease in TSH levels. The obtained results confirm the variety of adaptive mechanisms in the thyroid gland resulting from different iodine supplies.

Negative consequences of excessive iodine intake in the human organism are indicated in some studies [10, 40]. The incidence of thyrotoxicosis was increased following periods of mandatory salt iodization, compared with a period when supplementation was not required. The study conducted by Galofré et al. [10] determined the incidence rate of thyrotoxicosis before and during dietary-iodine supplementation in an iodine-sufficient area (Vigo, South Galicia, northwest of Spain). Dietary iodine supplementation in iodine-sufficient areas may induce an increase in the incidence of thyrotoxicosis. On the other hand, another iodine supplementation program in Bangladesh has shown no increased risk of thyroid dysfunction [32]. The study showed that mandatory mass iodination of table salt consumed in a hyper-endemic iodine-deficient area was safe and did not cause any side effects. Parveen et al. [32] suggest close regular monitoring of T3, T4, and TSH and further evaluation of any probable link between iodine-induced hypo- or hyperthyroidism and mass iodination of table salt in specifically designed studies. The number of reported cases of thyroid cancer, particularly papillary thyroid cancer, has also increased following iodine supplementation in some studies, including a nearly 20-year study in northeastern China [4] and an above 50-year study in Denmark [3].

A high level of iodine consumption leads to changes in the immunogenicity of the thyroglobulin molecules, changes in the regulation of intracellular adhesion in the vascular system, and generation of reactive oxygen species in the thyroid gland. Similar temporal effects can be observed with the use of iodized salt, which is used to prevent iodine deficiency diseases in almost all countries where iodine deficiency is registered [6, 11]. At the same time, it is considered that relatively low risks of excess iodine are far outweighed by the considerable risks of iodine deficiency [48]. Iodized salt programs need to be carefully monitored to ensure adequate iodine intake while avoiding iodine excess [47]. An increase in the HT incidence as a result of high levels of iodine intake can cause an increase in the incidence of endemic goiter. An increase in AITD due to high iodine intake may account for the increase in goiter prevalence. Fernando et al. [8] have assessed the prevalence of autoimmune thyroiditis after universal salt iodization in Sri Lanka. The results of this research proved that goiter prevalence increased after an initial drop following the ionization. A significant proportion of goiter is due to AITD. Urinary excretion of iodine in the community is high and has a positive correlation with the prevalence of TPO antibodies. Iodine-induced thyroid autoimmunity is related to TgAb. Latrofa et al. [23] have correlated iodine intake, thyroid autoimmunity, and recognition of thyroglobulin (Tg) epitopes after the implementation of iodine prophylaxis. Iodized salt use did not affect non-HT subjects; however, TgAb was more frequently detected in iodized salt users (IS-users) (18.9 vs. 13.6%, *p* = 0.02). Among subjects with Hashimoto’s thyroiditis, both positive TgAb (58.4 vs. 31.8%, *p* = 0.03) and TPOAb (61.5 vs. 45.4%. *p* = 0.04) were more frequent in IS-users [23]. Manousou et al. [26] have correlated the water iodine concentration to the urinary iodine concentration in a national survey of school-aged children. The water iodine concentration still contributes to iodine nutrition, but iodination overrides the goiter effect.

According to a survey conducted in Whickham, the mean prevalence of spontaneous hypothyroidism as a consequence of AITD was 3.5–5 per 1000 in women (mean age 57 years) and 0.6–1 per 1000 in men, respectively [35, 43]. Studies conducted by McLeod and Cooper [27] presented similar data from other geographical areas, i.e., Europe (UK, Spain, Italy, Germany, Slovenia, Nordic countries), North America (United States), Australia, and North Asia (Japan, China). In Poland, in 2006–2013, the prevalence of newly diagnosed HT dropped from 10.4 to 4.8% (*p* < 0.001) alongside with a decrease in the prevalence of newly diagnosed hypothyroidism from 17.8 to 7.7%. HT was prevalent among young women aged 20–39, and relatively more cases were recorded in the southern areas of Poland [12].

Over the past 10 years, the AITD incidence in Ukraine has increased by 68% and by 82% in terms of a 1000 population. The AITD prevalence ranges from 0.1 to 1.2% in children and from 6 to 11% in over 60-year-old women. The peak incidence of AITD falls on the working age and is 4–8 times more common in women than in men. However, today there is an upward trend in the incidence, especially in the younger age groups [22]. According to reports of the endocrinology service of Ukraine in 2014–2018, the mean 5-year HT incidence was 421.2 per 100,000 individuals in the whole population of Ukraine; 341.1 per 100,000 individuals in the population of the Lviv region; and 142.8, 188.3, 288.7, 279.5, and 168.4 per 100,000 individuals in other western regions of Ukraine, i.e., Volyn, Zakarpattia, Ivano-Frankivsk, Rivne, and Ternopil, respectively. Thus, the HT incidence in the Lviv region is the highest in the population of the western region of Ukraine.

The prevalence of hypothyroidism has been reported to be related to seasons. The effect of seasonal changes on the transition between subclinical hypothyroid and euthyroid status was demonstrated by Kim et al. [18]. The monthly distribution of follow-up TSH levels indicated a biphasic pattern, i.e., an increase during the winter-spring season and a decrease during the summer-fall season, with a maximal TSH difference of 0.69 mIU/L in subclinical hypothyroidism and 0.30 mIU/L in euthyroid subjects. Normalization of subclinical hypothyroidism was increased 1.4-fold in follow-up tests during the summer-fall follow-up, whereas subclinical hypothyroidism increased 1.4-fold in euthyroid subjects during the winter-spring follow-up [18]. The adaptive thermogenesis could be a reason for this seasonal variation in the TSH concentration [37]. Thus, the seasonal change should be considered in the interpretation of the TSH level and AITD in countries with substantial temperature differences between winter and summer [17].

## Conclusions

We have confirmed the hypothesis that the water iodine concentration is low, suggesting a weak and moderate degree of iodine endemia in the studied areas and that it is associated with the previous presence of goiter in the western regions of Ukraine. An increase in the water iodine concentration resulted in the increment in TgAb titers in both groups of females and males consuming water from the water supply network and in the TPOAb levels in the female group drinking water from the individual wells. An inverse correlation between the water iodine concentrations and the TSH levels was recorded in the female patients. TgAb and TPOAb were the independent factors associated with iodine concentration in water. This is important to consider during evaluation of current iodination programs for iodine prophylaxis (mass, group, or individual) in endemic regions. Besides the assessment of hormonal status, attention should be paid to the measurement of the urine iodine concentration in patients, especially in women.

## Data Availability

The data that support the findings of this study are available from the corresponding author, [Natalia Kurhaluk], upon reasonable request.

## References

[1] Allen EM, Appel MC, Braverman LE (1986). The effect of iodide ingestion on the development of spontaneous lymphocytic thyroiditis in the diabetes-prone BB/W rat. Endocrinology.

[2] Baretić M (2011). 100 godina Hashimotova tireoiditisa, bolesti koja jos uvijek intrigira – prikaz bolesnice [100 years of Hashimoto thyroiditis, still an intriguing disease]. Acta Med Croatica.

[3] Blomberg M, Feldt-Rasmussen U, Andersen KK, Kjaer SK (2012). Thyroid cancer in Denmark 1943-2008, before and after iodine supplementation. Int. J. Cancer.

[4] Dong W, Zhang H, Zhang P, Li X, He L, Wang Z, Liu Y (2013). The changing incidence of thyroid carcinoma in Shenyang, China before and after universal salt iodization. Med Sci Monit..

[5] Duntas LH (2011). Environmental factors and thyroid autoimmunity. Ann Endocrinol (Paris).

[6] Duntas LH (2015). The Role of Iodine and Selenium in Autoimmune Thyroiditis. Horm Metab Res.

[7] Ershow AG, Skeaff SA, Merkel JM, Pehrsson PR (2018). Development of Databases on Iodine in Foods and Dietary Supplements. Nutrients.

[8] Fernando RF, Chandrasinghe PC, Pathmeswaran AA (2012). The prevalence of autoimmune thyroiditis after universal salt iodisation in Sri Lanka. Ceylon Med J..

[9] Fuge R, Johnson CC (2015). Iodine and human health, the role of environmental geochemistry and diet: A review. Appl Geochem.

[10] Galofré JC, Fernández-Calvet L, Ríos M, García-Mayor RV (1994). Increased incidence of thyrotoxicosis after iodine supplementation in an iodine-sufficient area. J Endocrinol Invest..

[11] Hu S, Rayman MP (2017). Multiple Nutritional Factors and the Risk of Hashimoto's Thyroiditis. Thyroid.

[12] Jóźków P, Lwow F, Słowińska-Lisowska M, Mędraś M (2017). Trends in the prevalence of autoimmune thyroiditis in the leading private health-care provider in Poland. Adv Clin Exp Med..

[13] Kahaly GJ, Dienes HP, Beyer J, Hommel G (1998). Iodide induces thyroid autoimmunity in patients with endemic goitre: a randomised, double-blind, placebo-controlled trial. Eur J Endocrinol..

[14] Kasiyan OP, Pavliv RM, Khalay OA, Khabarova OV, Manenko AK (2007). Vyznachennya vmistu yodu u pytniy vodi endemichnoho rehionu Ukrayiny na prykladi Lvivskoyi oblasti [Determination of iodine content in drinking water of the endemic region of Ukraine on the example of Lviv region]. Practical Medicine (Praktychna medytsyna).

[15] Kasiyan OP, Pavliv RM, Khalay OA, Khabarova OV, Manenko AK (2008). Kilkisne vyznachennya kontsentratsiyi yodu v pytniy vodi endemichnoho rehionu Ukrayiny (Lvivs′ka oblast) [Quantitative determination of iodine concentration in drinking water of the endemic region of Ukraine (Lviv region)]. Practical Medicine (Praktychna medytsyna).

[16] Kasiyan OP, Manenko AK, Tkachenko HM, Pavliv RM, Khalay OA (2009). Zastosuvannya inversiynoho volt-amperometrychnoho metodu dlya vyznachennya vmistu yodu v pytniy vodi endemichnoho rehionu [Application of inversion volt-amperometric method for determination of iodine content in drinking water of the endemic region]. Hygiene of settlements (Hihiyena naselenykh misc).

[17] Kim YA, Park YJ (2014). Prevalence and risk factors of subclinical thyroid disease. Endocrinol Metab (Seoul.).

[18] Kim TH, Kim KW, Ahn HY, Choi HS, Won H, Choi Y, Cho SW, Moon JH, Yi KH, Park DJ, Park KS, Jang HC, Kim SY, Park YJ (2013). Effect of seasonal changes on the transition between subclinical hypothyroid and euthyroid status. J Clin Endocrinol Metab..

[19] Koukkou EG, Roupas ND, Markou KB (2017). Effect of excess iodine intake on thyroid on human health. Minerva Med..

[20] Kozyarin IP, Korzun VN (2009). Medyko-sotsialni problemy profilaktyky yododefitsytnykh zakhvoryuvan [Medico-social problems of prevention of iodine deficiency diseases]. The Mystetstvo likuvannya (Art of Treatment).

[21] Krassas GE, Rivkees SA, Kiess W (eds) (2007) Diseases of the Thyroid in Childhood and Adolescence. Pediatric and Adolescents Medicine, Basel, Karger, vol. 11:104–117

[22] Kravchenko VI, Postol SV (2011). Dynamika zakhvoryuvanosti na patolohiyu shchytopodibnoyi zalozy v Ukrayini. Mizhnarodnyy endokrynolohichnyy zhurnal, 35(3): 26-31. Kravchenko V.I., Postol S.V. 2011. Dynamics of disease on the pathology of thyroid disease in Ukraine. International Endocrinological Journal.

[23] Latrofa F, Fiore E, Rago T, Antonangeli L, Montanelli L, Ricci D, Provenzale MA, Scutari M, Frigeri M, Tonacchera M, Vitti P (2013). Iodine contributes to thyroid autoimmunity in humans by unmasking a cryptic epitope on thyroglobulin. J. Clin. Endocrinol. Metab..

[24] Laurberg P, Jørgensen T, Perrild H, Ovesen L, Knudsen N, Pedersen IB, Rasmussen LB, Carlé A, Vejbjerg P (2006). The Danish investigation on iodine intake and thyroid disease, DanThyr: status and perspectives. Eur J Endocrinol..

[25] Leung AM, Braverman LE (2014). Consequences of excess iodine. Nat. Rev. Endocrinol..

[26] Manousou S, Stål M, Eggertsen R, Hoppe M, Hulthén L, Filipsson Nyström H (2019). Correlations of water iodine concentration to earlier goitre frequency in Sweden-an iodine sufficient country with long-term iodination of table salt. Environ Health Prev Med..

[27] McLeod DS, Cooper DS (2012). The incidence and prevalence of thyroid autoimmunity. Endocrine.

[28] Method for measurements of the iodine concentration in drinking and mineral water, salt, bakery products, dairy products by the method of inversion voltammetry on a solid rotating electrode No. 081 / 12-0092-03; St. Petersburg, 2002. – 30 p.

[29] Mincer DL, Jialal II (2020) Hashimoto thyroiditis. StatPearls [Internet]. 2020 August. Available from: https://www.ncbi.nlm.nih.gov/books/NBK459262. 29083758

[30] Nystrӧm E, Berg GEB, Jansson SKG, Torring O, Valdemarsson SV (2011). Thyroid disease in adults.

[31] Oliynyk VA (1997). Endemichnyi zob [Endemic Goiter]. Likuvannia ta diagnostyka (Treatment and diagnosis).

[32] Parveen S, Latif SA, Kamal MM, Uddin MM (2007). Effects of long-term iodized table salt consumption on serum T3, T4, and TSH in an iodine deficient area of Bangladesh. Mymensingh Med J.

[33] Pearce EN (2014). Iodine deficiency in children. Endocr. Dev..

[34] Pedersen KM, Laurberg P, Nohr S, Jorgensen A, Andersen S (1999). Iodine in drinking water varies by more than 100-fold in Denmark. Importance for iodine content of infant formulas. Eur. J. Endocrinol..

[35] Ragusa F, Fallahi P, Elia G, Gonnella D, Paparo SR, Giusti C, Churilov LP, Ferrari SM, Antonelli A (2019). Hashimotos’ thyroiditis: epidemiology, pathogenesis, clinic and therapy. Best Pract Res Clin Endocrinol Metab.

[36] Schumm-Draeger PM (2004). Jod und thyreoidale Autoimmunität [Iodine and thyroid autoimmunity]. Z. Arztl. Fortbild Qualitatssich.

[37] Silva JE (2003). The thermogenic effect of thyroid hormone and its clinical implications. Ann. Intern. Med..

[38] Skalnyi AV, Kudrin AV (2000) Radiation, microelements, antioxidants, and immunity (trace elements and antioxidants in health improving). Mir, Moscow. Russian.

[39] Thomson CD, Campbell JM, Miller J, Skeaff SA (2011). Minimal impact of excess iodate intake on thyroid hormones and selenium status in older New Zealanders. Eur J Endocrinol.

[40] Todd CH, Allain T, Gomo ZA, Hasler JA, Ndiweni M, Oken E (1995). Increase in thyrotoxicosis associated with iodine supplements in Zimbabwe. Lancet.

[41] Tomer Y, Huber A (2009). The etiology of autoimmune thyroid disease: a story of genes and environment. J Autoimmun..

[42] Tronko M, Kravchenko V, Fink D, Hatch M, Turchin V, McConnell R, Shpak V, Brenner A, Robbins J, Lusanchuk I, Howe G (2005). Iodine excretion in regions of Ukraine affected by the Chornobyl Accident: experience of the Ukrainian-American cohort study of thyroid cancer and other thyroid diseases. Thyroid.

[43] Tunbridge WM, Evered DC, Hall R, Appleton D, Brewis M, Clark F, Evans JG, Young E, Bird T, Smith PA (1977). The spectrum of thyroid disease in a community: the Whickham survey. Clin. Endocrinol. (Oxf.).

[44] van den Briel-van Ingen T (2001) Iodine deficiency and functional performance of schoolchildren in Benin. Ph.D. Theses, Wageningen Universiteit, Wageningen, Netherlands, 2001, P. 160. Access: https://edepot.wur.nl/199106

[45] Williams DE, Le SN, Godlewska M, Hoke DE, Buckle AM (2018). Thyroid peroxidase as an autoantigen in Hashimoto’s disease: structure, function, and antigenicity. Horm Metab Res.

[46] Zar JH (1999). Biostatistical Analysis.

[47] Zimmermann MB (2013). Iodine deficiency and excess in children: worldwide status in 2013. Endocr Pract.

[48] Zimmermann MB, Jooste PL, Pandav CS (2008). Iodine-deficiency disorders. Lancet.

